# Visualization of the improvement of myocardial perfusion after coronary intervention using motorized fractional flow reserve pullback curve

**DOI:** 10.1007/s12928-016-0448-3

**Published:** 2016-12-10

**Authors:** Akiko Matsuo, Satoshi Shimoo, Kazuaki Takamatsu, Yumika Tsuji, Atsushi Kyodo, Kayoko Mera, Masahiro Koide, Koji Isodono, Yoshinori Tsubakimoto, Tomohiko Sakatani, Keiji Inoue, Hiroshi Fujita

**Affiliations:** Department of Cardiology, Japanese Red Cross Kyoto Daini Hospital, 355-5 Haruobi-cho, Marutamachi Kamannza-dori, Kamigyo-ku, Kyoto, 602-8026 Japan

**Keywords:** Fractional flow reserve, Motorized pullback curve, Coronary intervention

## Abstract

This study aimed to evaluate the feasibility and utility of using motorized pullback of the pressure guidewire to provide a graphic assessment and prediction of the benefits of coronary intervention. Fractional flow reserve (FFR) measurements were performed with motorized pullback imaging in 20 patients who underwent successful percutaneous coronary intervention (PCI) of the left anterior descending artery. Physiological lesion length (PLL) was calculated using frame counts to determine stent length. FFR area was calculated by integrating the FFR values recorded during pullback tracing (FFRarea). The percentage increase in FFR area (%FFRarea) was defined as the ratio of the difference between the pre- and post-intervention FFRarea to the total frame count. The average FFR values were enhanced following PCI, from 0.64 to 0.82, and the median value of the difference between pre- and post-interventional FFR values (D-FFR) and %FFRarea were 0.13 and 10.6%, respectively. The %FFRarea demonstrated a significant positive correlation with D-FFR (*R* ^2^, 0.61; *p* < 0.01). PLL tended to be longer and the %FFRarea was smaller in lesions with a gradual pressure-drop pattern than those with an abrupt pressure-drop pattern (35.37 vs. 20.40 mm, *p* = 0.07; 5.78 vs. 16.21%, *p* < 0.05, respectively). Motorized pullback tracing was able to identify the extent and location of stenosis and help in appropriate stent implantation, in addition to visualizing and quantifying the improvement in FFR following PCI.

## Background

**Fig. 1:**
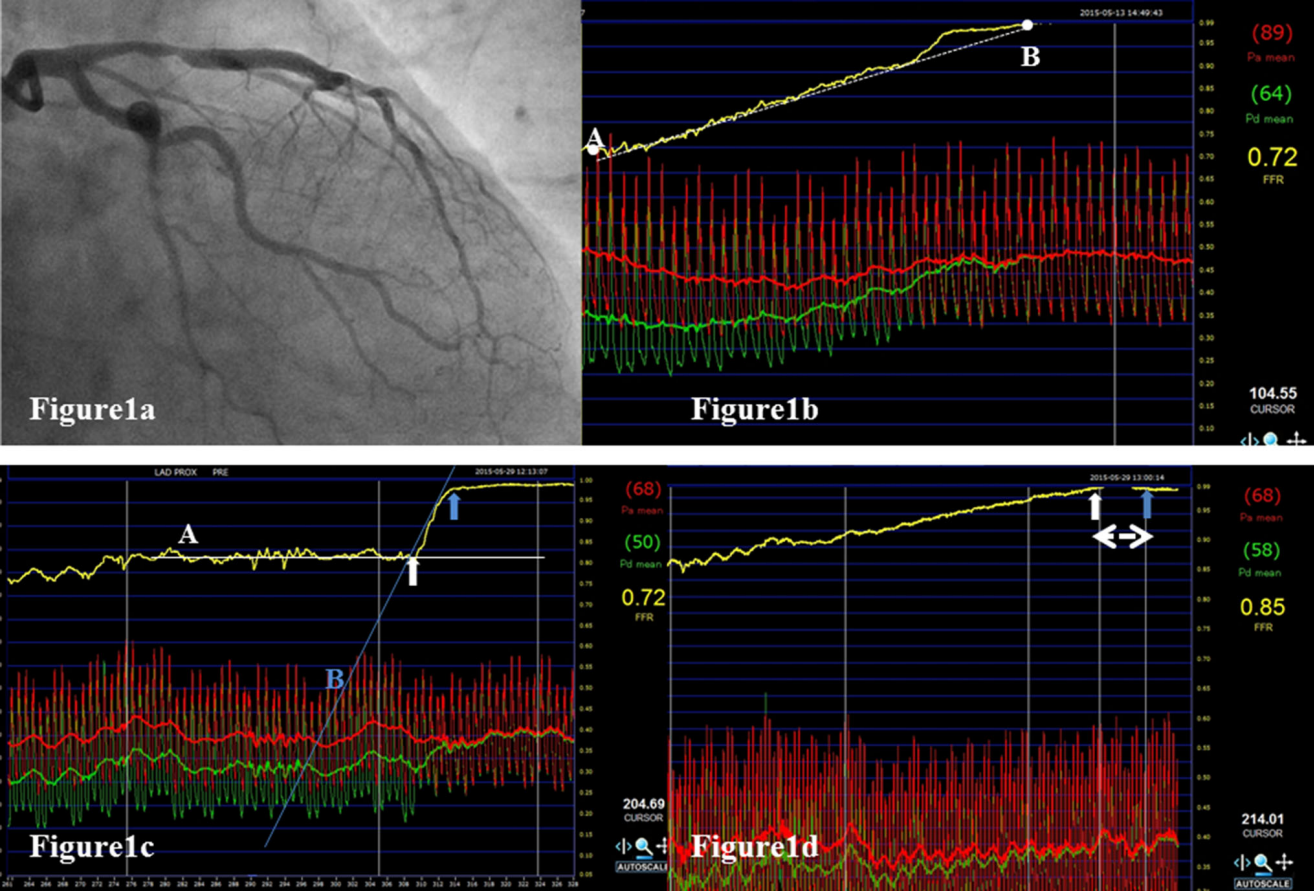
Angiogram and pullback coronary pressure tracing of a representative case. **a** Left coronary angiogram reveals intermediate stenosis at the proximal segment of the left anterior descending coronary artery (LAD). **b** Manual pullback pressure tracing in the LAD with intermediate stenosis demonstrates an FFR of 0.72 and a gradual pressure-drop pattern. The pullback curve is close to the line extending from point A to point B (*dotted line*). *Point A* is the pullback starting point. *Point B* is the first point where the FFR value reaches 1.0. **c** Motorized pullback pressure tracing in the same LAD demonstrates an abrupt drop of coronary pressure indicating the culprit lesion (*white* and *blue arrows*). The pullback curve has two different tangents (*white line A* and *blue line B*), forming an inflection point. The physiological lesion is defined as the difference between two inflection points (*white* and *blue arrow*). The ratio of Pd to Pa (Pd/Pa) at the distal and proximal inflection points is recorded as the distal and proximal Pd/Pa. The difference between the distal and proximal Pd/Pa was termed the lesion delta FFR. **d** Motorized pullback tracing following PCI with stent placement demonstrates an improved FFR of 0.85 with a gradual pressure-drop. The *double arrow* represents the stented lesion. The Pd/Pa at the distal (*white arrow*) and proximal (*blue arrow*) edges of the stent is demonstrated. Delta FFR in-stent (ΔFFR in-stent) was defined as the difference in the ratio between these two points. *LAD* left anterior descending coronary artery, *Pd* distal coronary pressure, *Pa* aortic pressure, *FFR* fractional flow reserve

Fractional flow reserve (FFR) is an invasive index and an established measure of the physiological severity of coronary stenosis. It is defined as the ratio of the maximal blood flow in the presence of a stenosis to the maximal blood flow in the absence of the stenosis. FFR is calculated by dividing distal coronary artery pressure (Pd) values by aortic pressure (Pa) values during maximal vasodilatation [1–3]. FFR is useful in determining the need for percutaneous coronary intervention (PCI), and studies have found better clinical outcomes with FFR-guided PCI [4–7]. FFR values obtained following PCI were found to be significantly related to repeated target vessel revascularization as well as death or acute myocardial infarction (AMI) [8–12]. This suggests that post-interventional FFR has the potential to identify whether the reduction in the pressure gradient following mechanical dilatation is adequate or not, which may improve the outcomes with PCI. However, such studies require accurate localization of the stenosis in the vessel. While the pullback curve facilitates the exact localization of the stenosis for the measurement of FFR, manual pullback of the pressure guidewire is limited by its lack of constant speed and may be unable to accurately localize the stenosis (Fig. 1b, c). Therefore, we introduced motorized pressure-wire pullback for a more consistent and reliable pressure tracing.

This study was performed to test the hypothesis that motorized pressure-wire pullback is feasible in visualizing and quantifying the improvement in FFR following PCI.

## Methods

### Study population

The study included 20 patients with intermediate coronary lesions in the left anterior descending (LAD) artery as identified on coronary angiography. Included patients presented with chest pain and had evidence of ischemic changes on an exercise test, single-photon emission computed tomography (SPECT), or ambulatory electrocardiography. Patients with shock, totally occlusive lesions, severe tortuous lesions, tandem lesions, multivessel disease, old myocardial infarction (OMI), congestive heart failure, acute coronary syndrome, or those receiving hemodialysis were excluded. The protocol was approved by the ethics committee of our hospital, and all patients provided written, informed consent.

### Intracoronary measurements and pressure-wire pullback

Intracoronary nitroglycerin (200 µg) was administered in all the cases before the introduction of pressure wires. Intracoronary pressure measurements were performed using a 0.014-in coronary pressure sensor-tipped Aeris wire (St. Jude Medical, USA). Pressure-wire pullback was performed at rest in a mechanized manner using a pullback device of a scanning-type intravascular ultrasound system (R100, Volcano Corp. USA). Pharmacological hyperemia was induced by intravenous administration of adenosine triphosphate (ATP) at a rate of 150 µg/min via the median cubital vein, and the rate was increased to 180 µg/min to achieve steady-state hyperemia. The guidewire was then advanced through the lesion and positioned not farther than 12 cm from the ostium. The proximal edge of its radiopaque portion was positioned at the ostium of the distal branch as a landmark of the starting point for pullback. Pullback was started at a speed of 1 mm/s during maximum hyperemia and continued until the pressure sensor reached the left main stem ostium. FFR was calculated as the ratio of Pd to Pa at the starting point. PCI was performed for lesions with FFR values of 0.8 or less, followed by post-interventional measurement of FFR with the mechanized pressure-wire pullback. PCI was aimed at achieving post-interventional FFR values greater than 0.8 to the extent possible.

Patients were divided into two groups according to the pattern of pullback coronary pressure tracing: the abrupt pressure-drop pattern (Abrupt) and the gradual pressure-drop pattern (Gradual), depending on the loss of pressure along the arterial length [13]. In this study, these two patterns were defined as follows: the gradual pressure-drop pattern is characterized by a pullback curve that is close to the line passing through the start point (Fig. 1b, point A) and the first point where the FFR value returned to 1.0 (Fig. 1b, point B); the abrupt pressure-drop pattern is characterized by a pullback curve with more than two different tangential lines forming an inflection point (Fig. 1c, white line A, blue line B). Among patients with the gradual pressure-drop pattern, the target lesion for stenting was defined at the discretion of each operator.

### Calculation of indices in pullback tracing analysis

**Fig. 2:**
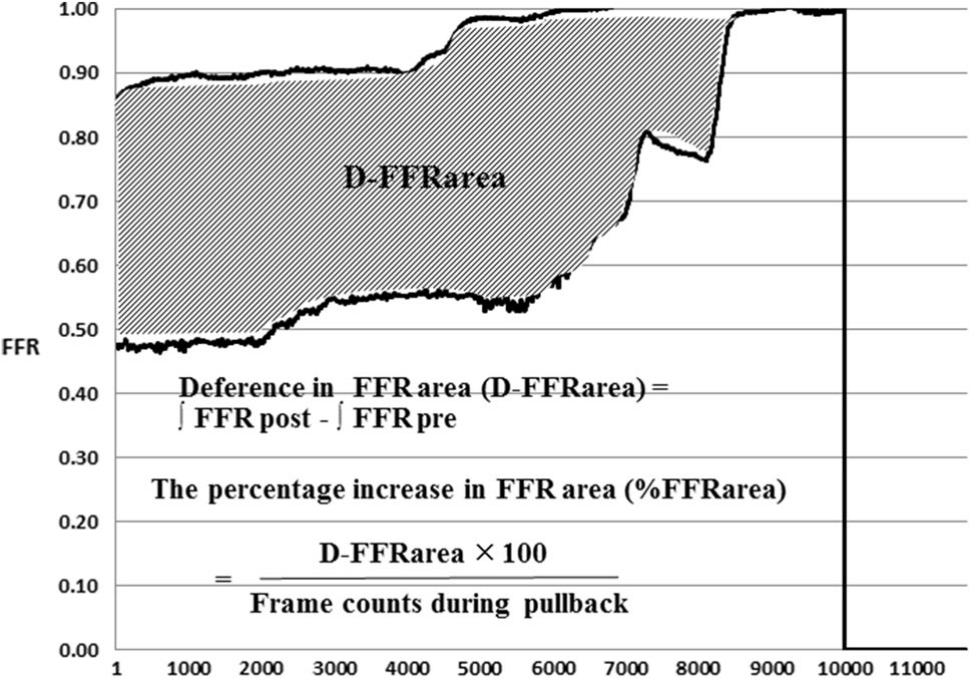
Quantitative analysis of pre- and post-interventional mechanized pullback tracing. The *vertical axis* shows the FFR values and the *horizontal axis* shows the frame rates. The *area under each curve* is calculated by integration and the difference in the pre- and post-intervention FFR area is calculated to give the D-FFR area (*hatched area*). The percentage increase in the FFR area (%FFRarea) is defined as the D-FFR area divided by the number of frame counts during the pressure-wire pullback. These parameters are calculated in an Excel spread sheet

Figure 1b shows the pre-interventional FFR pullback tracing achieved by the mechanical auto-pullback system. The physiological lesion was defined based on the difference between two inflection points on pullback tracing, while the physiological lesion length (PLL) was calculated based on the frame counts (frame rate: 100 frame per second). The ratios of Pd to Pa (Pd/Pa) at the distal and proximal inflection points were recorded as the distal and proximal Pd/Pa. The difference between the distal and proximal Pd/Pa was calculated as the lesion delta FFR (lesion ΔFFR) (Fig. 1b). Similarly, in the post-intervention pullback tracing, Pd/Pa at the distal and proximal edge of the stent was recorded, and delta FFR in-stent (ΔFFR in-stent) was defined as the difference in the ratio between these two points (Fig. 1c). Figure 2 shows pre- and post-intervention mechanized pullback tracings, with all measurements during pullback being exported to a spreadsheet (Microsoft Excel 2010) for visualizing and quantifying the improvement in FFR following intervention. The area under each pullback curve was calculated by integration and defined as the pre- and post-intervention FFR area. The pre- and post-intervention difference in the FFR area was defined as the D-FFR area. The percentage increase in the FFR area (%FFR area) was defined as the D-FFR area divided by the frame counts during the pressure-wire pullback (Fig. 2).

### Quantitative coronary angiography (QCA)

QCA was performed by an operator blinded to the results of FFR using a validated, automated edge-detection software (CCIP-310/W; Cathex, Tokyo, Japan). Minimum lumen diameter (MLD), reference diameter (RD), lesion length, and percent diameter stenosis (%DS) were measured.

### Intravascular images and analysis

Intravascular ultrasound (IVUS) assessments were performed using a commercially available system (Terumo, Tokyo, Boston Scientific, Natick, Massachusetts). A frequency-domain optical coherence tomography (OCT) system (C7-XR OCT Intravascular Imaging System, St Jude Medical, St. Paul, MN) or optical frequency-domain imaging (OFDI) system (LUNAWAVE, Terumo Corp., Tokyo, Japan) was used. The choice of imaging device was made at the operator’s discretion. The quantitative evaluation of intravascular images included the minimal lumen area (MLA), proximal and distal reference (ref) lumen area, lesion length (LL), and minimal stent area (MSA). Heavy calcified plaque was defined as plaque with a cross-sectional calcium arc greater than 180°. Lipid-rich plaque was defined as a lipidic arc greater than 180°. Lipidic plaque on OCT or OFDI images was identified as a signal-poor lipid pool with poorly delineated borders beneath a homogeneous signal-poor band. These were identified on IVUS images as attenuated and echolucent plaques.

### Myocardial perfusion imaging

Stress-rest myocardial perfusion studies were performed for 12 of 20 patients (60%) using technetium-99-m tetrofosmin. Pharmacological stress test was performed using ATP (140 mg/min). Acquisition and processing protocols used for Tc-99-m tetrofosmin SPECT studies have been described in detail previously [14]. For SPECT interpretation, the “summed stress score” and “summed rest score,” were evaluated. The “summed difference score” (SDS) was defined as the difference between the summed stress score and the summed rest score [14]. This scoring system was used in the subanalysis.

### Statistical analyses

A commercially available statistical package (SPSS 13.0, SPSS Inc., Chicago, IL, USA) was used for data analyses. Continuous variables were expressed as the mean and standard deviation and were compared using the unpaired *t* test or Mann–Whitney *U* test. Pearson’s correlation was used to estimate a linear relationship between two quantitative variables. Linear regression analyses were carried out to assess univariate relationships between continuous variables. A value of *p* < 0.05 was considered statistically significant.

## Results

### Practicality and safety of the mechanical auto-pullback system

We conducted repeated motorized pullback trials in vitro to verify the accuracy and reproducibility of the pullback speed. The root-mean-square error and standard error were found to be 0.020 and 0.0002, respectively (*n* = 50). After the in vivo examination, mechanical auto-pullback was performed in all 20 patients without any complications. In all 20 patients, pre-interventional FFR was found to be less than 0.8 (average: 0.64 ± 0.11) and PCI was able to be successfully performed. The residual stenosis was less than 30% in all patients (average: 8.4 ± 16.01%), and there were no complications. The average post-interventional FFR was 0.82 ± 0.05. However, the optimal post-intervention FFR (>0.8) could not be achieved in two patients despite repeated dilatation. The reason for stent under expansion in one patient was confirmed as heavy calcification on OFDI. No reason could be established in the second patient, who had a minimal stent area of 5.3 mm^2^.

### Comparison of clinical characteristics between the abrupt and gradual groups

There were 13 patients in the abrupt group and 7 in the gradual group. Baseline clinical and angiographic characteristics of the two groups are presented in Table 1. There were no significant differences between the two groups, except that post-interventional %DS was significantly lower in the gradual group than in the abrupt group (−2.73 ± 8.43 vs. 14.39 ± 16.75%, *p* < 0.05) (Table 1).

**Table 1:** Clinical and angiographic characteristics

	Abrupt, *n* = l3	Gradual, *n* = 7	
Age	71.3 ± 12.2	71.9 ± 19.0	0.92
Prior MI. *n* (%)	5 (38.4)	2 (28.6)	0.95
Male	11 (84.6)	6 (85.7)	0.66
Diabetic mellitus	4 (30.8)	4 (57.1)	0.25
Family history	3 (23.0)	2 (28.6)	0.79
Dyslipidemia	12 (92.3)	3 (42.8)	0.95
Hypertension	10 (76.9)	4 (57.1)	0.36
Smoking	7 (53.8)	7 (100.0)	0.10
Chronic kidney disease	5 (38.5)	3 (42.8)	1.00
Proximal lesion	12 (92.3)	6 (85.7)	1.00
Type B2/C	10 (76.9)	6 (85.7)	1.00
Calcified lesion	4 (30.8)	2 (28.6)	1.00
RD, mm	2.65 ± 0.41	2.82 ± 0.59	0.43
%DS; %	60.15 ± 16.89	53.81 ± 13.32	0.40
MLD, mm	1.07 ± 0.52	1.27 ± 0.32	0.38
Lesion length	19.02 ± 8.63	25.04 ± 12.59	0.22
Post-interventional MLD	2.89 ± 2.48	2.74 ± 0.44	0.85
Post-interventional %DS, %	14.39 ± 16.75	−2.73 ± 8.43	0.02
Stent diameter, mm	2.93 ± 0.30	2.93 ± 0.34	0.98
Stent length	26.92 ± 13.33	28.57 ± 15.72	0.81
Multiple stenting, *n* (%)	4 (30.8)	4 (57.1)	0.50

*MI* myocardial infarction, *RD* reference diameter, *%DS* percent diameter stenosis, *MLD* minimal lumen diameter

Intravascular imaging was performed using OFDI/OCT or IVUS, in 10 patients each. The plaque morphology was found to be similar in the two groups, although MLA tended to be larger in the gradual group than in the abrupt group (2.37 ± 0.49 vs 1.75 ± 0.72 mm^2^, *p* = 0.06) (Table 2a).

**Table 2:** Comparison of intravascular imaging and intracoronary measurements between two pressure-recovery patterns

	Abrupt, *n* = l 3	Gradual, *n* = 7	
(a) Intravascular images parameters			
Lipid-rich plaque, *n* (%)	8 (61.5)	6 (85.7)	0.95
Calcified plaque	5 (38.5)	1 (14.8)	0.39
Plaque rupture	4 (30.8)	0 (0.0)	0.22
Lesion length, mm	28.34 ± 14.64	27.31 ± 14.97	0.89
Proximal ref. lumen area, mm^2^	6.78 ± 1.81	7.98 ± 14.97	0.32
Distal ref. lumen area	5.42 ± 1.62	7.98 ± 14.97	0.11
Minimal lumen area	1.75 ± 0.72	2.37 ± 0.49	0.06
Minimal stent area	4.94 ± 1.55	5.61 ± 3.18	0.12
(b) Intracoronary pressure parameters			
Pre-interventional FFR	0.59 ± 0.11	0.74 ± 0.03	<0.01
Distal Pd/Pa	0.66 ± 0.12	0.78 ± 0.03	0.02
Proximal Pd/Pa	0.94 ± 0.05	0.93 ± 0.07	0.31
Physiological lesion length, mm	20.36 ± 14.53	35.37 ± 20.40	0.07
Lesion AFFR	0.29 ± 0.15	0.15 ± 0.05	0.01
Post-interventional FFR	0.81 ± 0.05	0.84 ± 0.02	0.19
AFFR in-stent	0.07 ± 0.05	0.05 ± 0.03	0.37
Pd/Pa at the proximal stent edge	0.92 ± 0.05	0.95 ± 0.03	0.46
Pd/Pa at the distal stent edge	0.88 ± 0.05	0.89 ± 0.05	0.91
Frame count	8837.8 ± 1934.2	11234.0 ± 764.5	<0.01
Pre-interventional FFRarea	6554.7 ± 1576.0	9634.0 ± 687.0	<0.01
Post-interventional FFRarea	7987.3 ± 1747.4	10275.5 ± 735.8	<0.01
%FFRarea	16.21 ± 8.86	5.78 ± 3.90	<0.01
D-FFR	0.22 ± 0.10	0.10 ± 0.03	<0.01

ref., reference; FFR, fractional flow reserve; Pd/Pa, the ratio of the mean distal coronary pressure to the mean aortic pressure; distal Pd/Pa, Pd/Pa at the distal portion of the lesion before intervention; proximal Pd/Pa, Pd/Pa at the proximal portion of the lesion before intervention; Lesion ΔFFR, the difference in Pd/Pa between the distal and proximal end of the lesion; ΔFFR in-stent, the difference in Pd/Pa between proximal and distal edge of the stent; FFR area, the area under the pullback curve of FFR calculated by integration; %FFRarea, The percentage gain in the FFR area; D-FFR, the difference in the pre- and post-intervention FFR values

### Comparison of physiological measurements between the abrupt and gradual groups

**Fig. 3:**
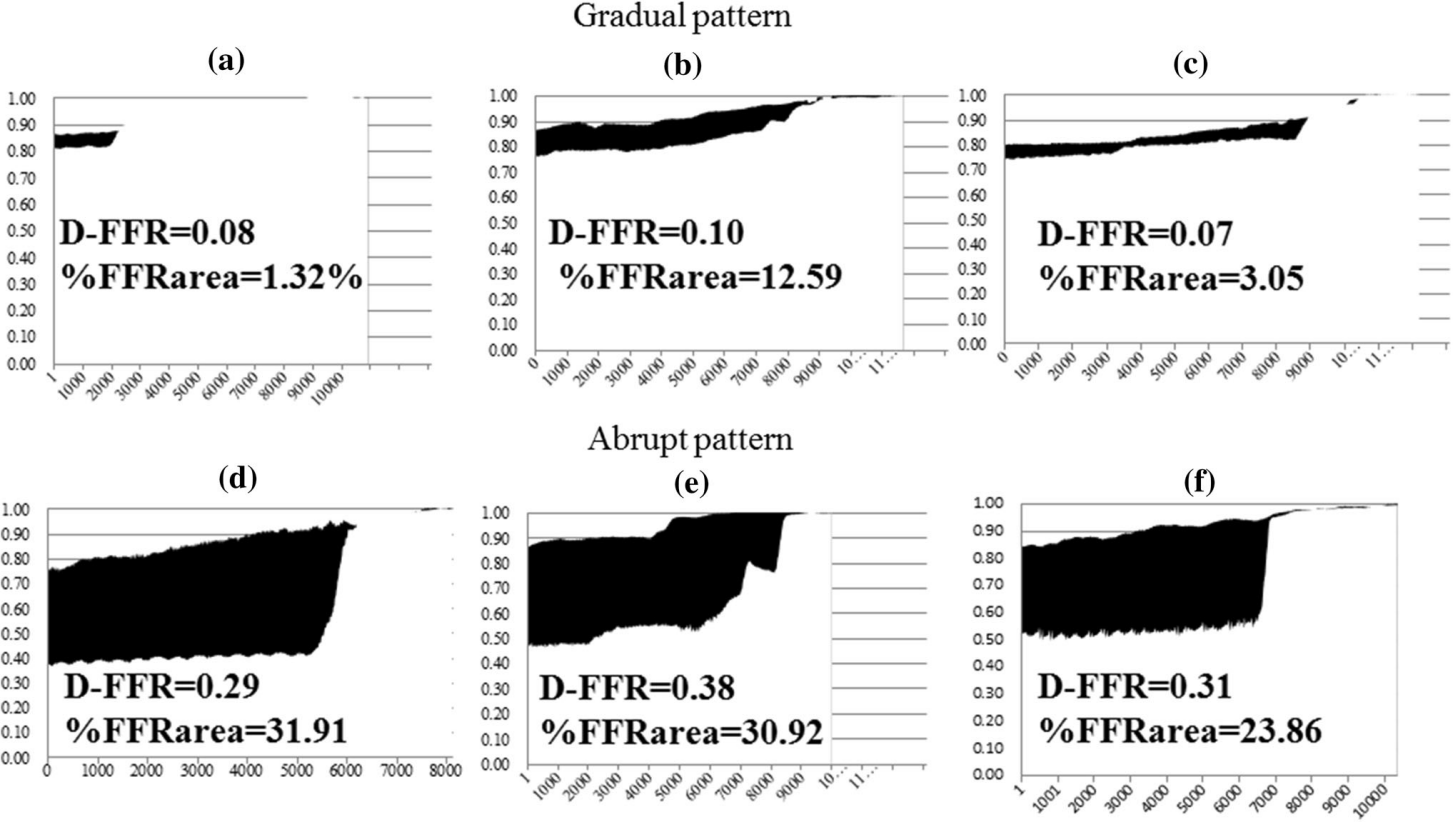
Representative composite graph of pullback tracing in representative patients with diffuse and abrupt patterns. The *blackened area* in the composite graph of auto-pullback tracing represents the difference between pre- and post-intervention FFR area (D-FFR area). **a**–**c** Pullback tracing in patients with a gradual pressure-drop pattern. **d**–**f** Pullback tracing in patients with an abrupt pressure-drop pattern. *D-FFR* the difference in pre- and post-intervention fractional flow reserve (FFR), *%FFRarea* the percentage increase in the FFR area (%FFRarea)

Representative composite graphs of pullback tracing among the patients with gradual and abrupt patterns are depicted in Fig. 3. The upper three graphs represent examples of the gradual group and the remaining are examples of the abrupt group. The gradual group had a smaller %FFR area with smaller D-FFR compared to the abrupt group (Fig. 3).

**Fig. 4:**
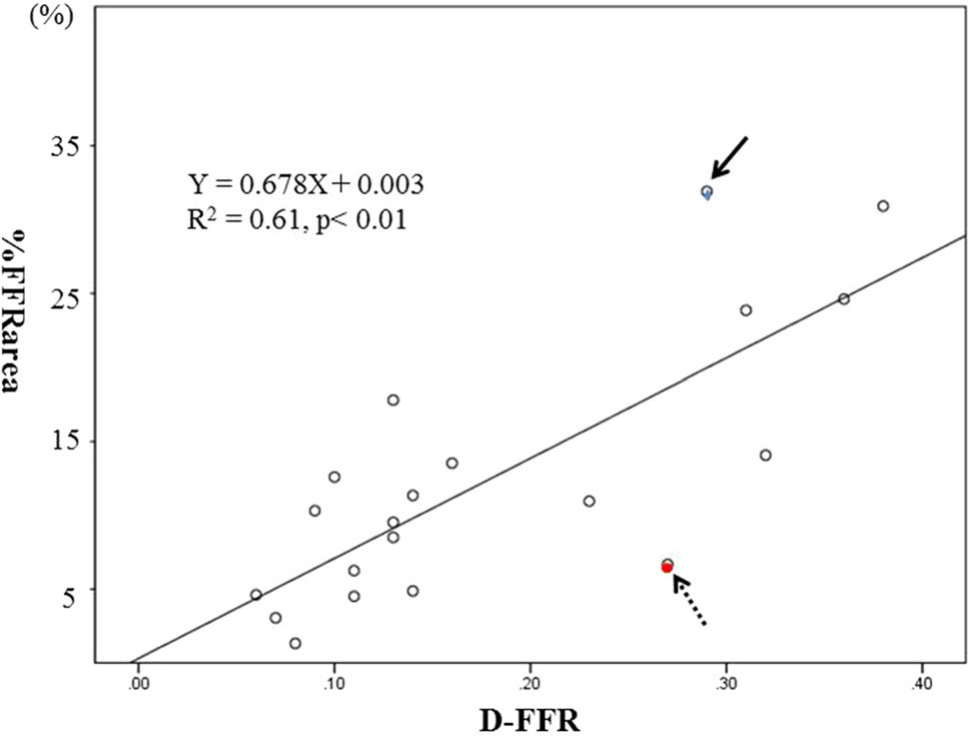
Relationship between the percentage increase in FFR area and pre- and post-interventional FFR difference. The %FFRarea values correlate positively with D-FFR (*r* = 0.782, *R* ^2^ = 0.612, *p* < 0.01). *%FFRarea* the percentage increase in the FFR area, *D-FFR* the pre- and post-intervention difference in FFR values. The *blue circle with a solid arrow* represents the case whose findings are presented in Fig. 3d. The *red circle with a dashed arrow* represents the case whose findings are presented in Fig. 5

Detailed physiological indices are presented in Table 2b. Pre-interventional FFR and Pd/Pa at the distal portion of the lesion were significantly higher in the gradual group than in the abrupt group (0.74 ± 0.03 vs. 0.59 ± 0.11, *p* < 0.01; 0.78 ± 0.03 vs. 0.66 ± 0.12, *p* < 0.05). Lesion ΔFFR (0.15 ± 0.05 vs. 0.29 ± 0.15, *p* < 0.05) was significantly smaller, while PLL (35.37 ± 20.40 vs. 20.36 ± 14.53 mm, *p* = 0.07) tended to be longer in the gradual group than in the abrupt group. Pre-interventional FFR area (9634.0 ± 687.0 vs. 6554.7 ± 1576.0, *p* < 0.01) and post-interventional FFR area (10275.5 ± 735.8 vs. 7987.3 ± 1747.4, *p* < 0.01) were significantly greater in the gradual group than in the abrupt group. However, considering the significant difference in the frame count between the gradual group and the abrupt group (11234.0 ± 764.5 vs. 8837.8 ± 1934.2, *p* < 0.01), %FFRarea (5.78 ± 3.90% vs. 16.21 ± 8.86%, *p* < 0.01), as well as D-FFR (0.10 ± 0.03 vs. 0.22 ± 0.10, *p* < 0.01) were significantly smaller in the gradual group than in the abrupt group. On analyzing the relationship between %FFRarea and D-FFR, the median values of %FFRarea and D-FFR were 10.63 and 0.13%, respectively. %FFRarea was found to have a significant positive correlation with D-FFR (*R* ^2^ = 0.61, *p* < 0.01) (Fig. 4).

With regard to objective myocardial ischemia, we performed a subanalysis of adenosine triphosphate stress myocardial perfusion imaging including 12 of 20 patients (gradual group, *n* = 3; abrupt group, *n* = 9). The average SDS in the gradual group was significantly less than that in the abrupt group (0.4 ± 0.6 vs. 2.2 ± 1.3, *p* = 0.04). Pearson’s correlation coefficient was found to be 0.45 (*p* = 0.10) between SDS and %FFRarea, showing a slight tendency toward a positive correlation. On the other hand, there was no correlation between SDS and D-FFR (*r* = −0.06, *p* = 0.84).

## Discussion

The use of a mechanized auto-pullback tracing of intracoronary pressure contributed to the following advantages: identification of the culprit lesion responsible for the pressure loss in the evaluated vessel; distinguishing the pattern of pressure-drop in pullback tracing as abrupt or gradual; identifying the difference in the area under the pullback curve between pre- and post-intervention studies to detect improvement in FFR values following PCI; detecting a smaller FFR improvement in the gradual group than the abrupt group; and establishing a positive correlation between the %FFRarea and D-FFR.

### Visualization of the benefit of coronary intervention

In this study, mechanized pressure-wire pullback was performed using an R100 Volcano Pullback device, which is the same system used in the recent report by Nijjer et al. [15]. In addition, we conducted repeated motorized pullback trials in vitro to verify the accuracy and reproducibility of the pullback speed. Therefore, pullback tracings with the motorized pullback device in this study were considered valid. A representative case is shown in Fig. 1. Manual pullback in the LAD artery, where there was an angiographic intermediate stenosis at the proximal portion (Fig. 1a), revealed a gradual pressure-drop pattern (Fig. 1b). However, the motorized pullback curve revealed a definite big step up in intracoronary pressure at the proximal portion of the LAD in the same patient (Fig. 1c), which was improved by stent implantation (Fig. 1d). In addition, another issue is suboptimal post-interventional FFR in spite of angiographic success. We reported that patients with post-interventional FFR <0.8 accounted for approximately 10%, regardless of whether mechanical intervention was added [12]. In these lesions, judging from the absolute FFR values, it follows that PCI provides no benefit for patients. Nevertheless, we expect some benefit from PCI, which relieves focal pressure loss. Thus, we tried to use mechanized pullback tracing to measure pre- and post-interventional FFR to visualize any benefit of PCI. The difference between the pre- and post-intervention integrated FFR values could be clearly expressed as a specific area in this study. This area was further expressed as a proportion of an expected post-interventional FFR value of 1.0, defined as the %FFRarea, allowing a visual representation of the quantitative improvement in FFR following PCI. This study only interrogated the LAD artery, to maintain consistent basal subtended myocardial perfused territories, which depend on the coronary arteries. In addition, LAD perfusion has a high clinical impact.

**Fig. 5:**
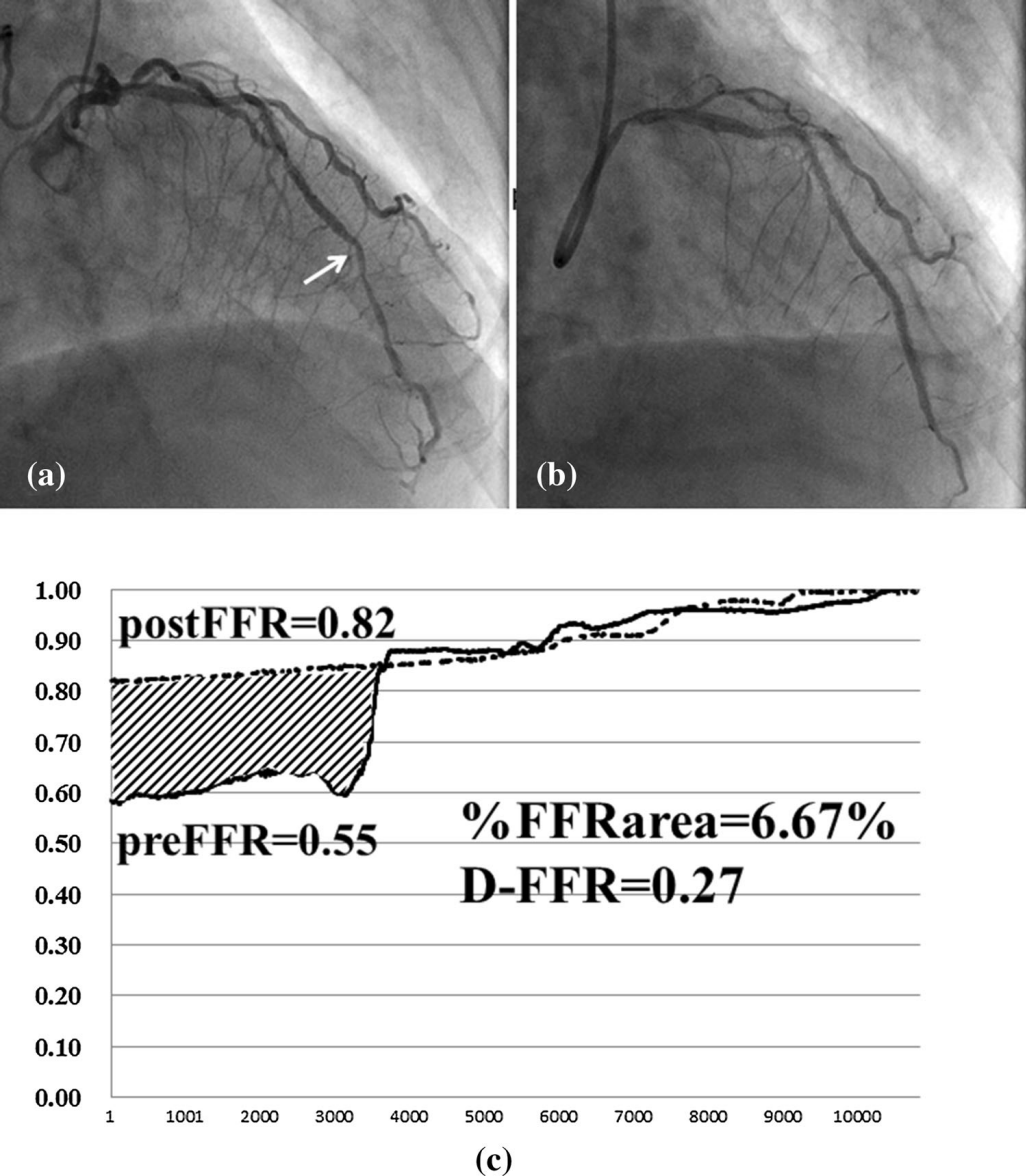
Coronary angiogram and composite graph. **a** Pre-intervention left coronary angiogram shows an intermediate lesion (*white arrow*) in the distal portion of the left anterior descending artery. Pre-interventional fractional flow reserve (FFR) was 0.55. **b** Post-intervention left coronary angiogram demonstrates the absence of a narrowing lesion following stent implantation. Post-interventional FFR was 0.82. **c** Pre- and post-interventional physiological maps using mechanized auto-pullback tracing. The percentage increase in the FFR area was 6.67%, while the difference in FFR value between pre- and post-intervention was 0.27. *preFFR* pre-interventional fractional flow reserve, *postFFR* post-interventional fractional flow reserve, %*FFRarea* the percentage increase in the FFR area, *D-FFR* the difference in pre- and post-intervention FFR values

On analysis of pressure tracings, the %FFRarea demonstrated a significant positive correlation with D-FFR. The advantages of measuring %FFRarea compared to recording the FFR values alone are illustrated by the findings pertaining to a case in this study (Fig. 3d). This figure presents the pullback tracings of a patient who had a post-interventional FFR of only 0.72 in spite of angiographic PCI success. Post-interventional IVUS in this patient revealed an MSA of 5.3 mm^2^, with no room to perform additional PCI. On diagrammatic representation, %FFRarea was calculated as 31.91% and corresponded to a D-FFR of 0.29. This amounts to a large area, as demonstrated in Fig. 3d. While the post-interventional FFR of 0.72 appeared to suggest that the revascularization was a physiological failure, %FFRarea provided diagrammatic evidence of the extent of physiological improvement following PCI. Conversely, in another case with a distally located lesion (Fig. 5) and a pre-interventional FFR of 0.55 (Fig. 5a), stent implantation resulted in a post-interventional FFR of 0.82 (Fig. 5b). Despite the large gain in FFR following the procedure, %FFRarea was small, calculated as 6.67% (Fig. 5c). Furthermore, an initial nuclear stress test did not show any reversible perfusion defects in this case, despite typical exertional chest oppression. This could be attributed to the small perfusion territory subtended by this stenosis. Furthermore, based on SPECT scoring subanalysis, there was a slight tendency of a positive correlation between SDS and %FFRarea, which was not statistically significant because of the small sample size. On the other hand, there was no relationship between SDS and D-FFR, which indicates the difference in two point values. Strictly speaking, SDS does not indicate the extent of improvement of myocardial ischemia by PCI, because PCI cannot salvage all myocardial ischemia, and the difference between pre- and post-SDS more ideally represents the extent of improvement of myocardial ischemia than the pre-intervention SDS. However, to date, there has been no research on the absolute quantification of improvement of myocardial ischemia pre and after PCI. In fact, we were not pragmatically able to perform SPECT studies both pre- and post-PCI.

### Clinical implications

Among the patients classified as having a gradual pressure-drop, myocardial ischemia could not be detected on SPECT images due to the continuous loss of pressure from the base to the apex [16]. The subanalysis showed that the gradual group had significantly less SDS than the abrupt group. In patients with a gradual pressure-drop pattern, the visualized %FFRarea was smaller than that in patients with an abrupt pressure-drop pattern. In other words, the benefit of PCI was lesser in lesions causing a gradual pressure-drop pattern. In these patients, a continuous loss of pressure along the arterial length corresponds to the graded base-to-apex perfusion abnormality, as demonstrated by positron emission tomography (PET), which indicated obvious myocardial ischemia that SPECT images could not detect [16]. Despite the presence of objective ischemia, there are no segmented stenotic lesions to dilate mechanically in these patients, and the abnormal resistance occurs due to the diffuse atherosclerotic epicardial coronary arteries [17], and therefore, mechanical luminal dilatation, as in PCI, is considered minimally effective. This may support the finding that the %FFRarea in patients with a gradual pressure-drop pattern was smaller than that in patients with an abrupt pressure-drop pattern. Moreover, in lesions located in the distal portion of the vessels, %FFRarea was found to be small, as aforementioned (Fig. 5), in spite of a lesion with an abrupt pressure-drop pattern. Hachamovitch reported that while revascularization had greater survival benefits compared to medical therapy in patients with moderate-to-large amounts of inducible ischemia, there were no additional benefits in patients with mild ischemia [18]. Therefore, ischemia-guided revascularization is an important element in current practice [18–23]. In this context, although auto-pullback of FFR might be advantageous, as comprehensible diagrammatic representation of pullback tracings aid in appropriate revascularization procedures for stenotic lesions at the distal portion or in patients with diffuse pressure-drop, further investigation including collection of data and introduction of measurements of the absolute coronary blood flow is required to confirm the definitive advantage of %FFRarea.

### Limitations

This study included only a small number of patients and there was no control group. In terms of the pressure-drop patterns, the classification of patients as gradual or abrupt was not defined in a completely quantitative manner, although the definitions were created using motorized pullback tracing. This study was based on the assumption that the motorized pullback tracing was accurate, as reported by Nijjer et al. [15]. We performed repeated tests to determine the accuracy of the pullback speed in vitro. However, even though severe tortuous and narrowing lesions were excluded, motorized pullback tracing cannot work in vivo in the exact same manner as it was verified in vitro. The pullback device limited the pullback length to a distance of only 12 cm in the interrogated vessel. Fluctuation of intracoronary pressure may have occurred owing to the use of peripheral intravenous administration of ATP, which may have affected the quality of the pullback tracings. Analysis of auto-pullback data, which was performed offline, took some time during the procedure. In addition, setting up and using the device was slightly cumbersome. The %FFRarea was analyzed as if it provided a virtual quantitative representation of the amount of ischemic myocardium, though it was not compared with the absolute myocardial blood volume subtended by the stenosis. We performed the SPECT subanalysis using a scoring system in only 60% of the patients. Other patients did not undergo SPECT scanning, because they had other evidence of myocardial ischemia. Therefore, this study did not have sufficient statistical power to determine the relationship between %FFRarea and SDS. Determining an accurate estimation of absolute coronary flow is challenging. It may have been preferable to analyze %FFRarea in comparison with cardiac PET images, because PET assesses absolute myocardial blood flow [24, 25], while both SPECT and magnetic resonance images only represent coronary flow reserves, and their results are semi-quantitative [26, 27].

Tandem lesions were excluded in the current study. The fluid dynamic interaction between the stenoses alters their relative severity and complicates the determination of the FFR for each stenosis separately, as opposed to using the simple ratio of Pd/Pa for a single stenosis. Hyperemic flow through one stenosis is limited by the presence of the other stenosis, and vice versa. Because hyperemic flow declines significantly if any 50% reduction in lumen diameter due to coronary intervention is observed, even mild secondary lesions can affect hyperemic pressure-only indexes. Therefore, intervention to remove a stenosis will increase hyperemic flow, which alters the significance of secondary lesions [15, 28]. The post-interventional pullback curve, which is measured under a hyperemic state after removal of one stenosis, will differ from the expected shape of a pullback curve assuming that one pressure gradient is simply removed. Thus, the %FFRarea in patients with tandem lesions is not a surrogate marker of improvement of ischemic myocardium.

## Conclusion

Pullback tracing using motorized pullback of the pressure guidewire was able to accurately identify the extent and location of stenosis. The pattern of pressure-drop on pullback tracing and the potential benefits of coronary intervention were also comprehensively demonstrated by the auto-pullback tracing. We hope that the present method would facilitate decision-making with respect to appropriateness of coronary intervention in patients with coronary artery disease.

## References

[1] Pijls NH, Van Gelder B, Van der Voort P, Peels K, Bracke FA, Bonnier HJ (1995). Fractional flow reserve a useful index to evaluate the influence of an epicardial coronary stenosis on myocardial blood flow. Circulation.

[2] Pijls NH, De Bruyne B, Peels K, Van Der Voort PH, Bonnier HJ, Bartunek J, Kolen JJ (1996). Measurement of fractional flow reserve to assess the functional severity of coronary artery stenosis. N Engl J Med.

[3] Hau WK (2004). Fractional flow reserve and complex coronary pathologic condition. Eur Heart J.

[4] Pijls NH, van Schaardenburgh P, Manoharan G, Boersma E, Bech JW, van’t Veer M (2007). Percutaneous coronary intervention of functionally nonsignificant stenosis: 5-year follow-up of the DEFER study. J Am Coll Cardiol.

[5] Tonino PA, De Bruyne B, Pijls NH, Siebert U, Ikeno F, van’ t Veer M (2009). Fractional flow reserve versus angiography for guiding percutaneous coronary intervention. N Engl J Med.

[6] De Bruyne B, Pijls NHJ, Kalesan B, Barbato E, Tonino PA, Piroth Z (2012). Fractional flow reserve–guided PCI versus medical therapy in stable coronary disease. N Engl J Med.

[7] Nam CW, Mangiacapra F, Entjes R, Chung IS, Sels JW, Tonino PA (2011). Functional SYNTAX score for risk assessment in multivessel coronary artery disease. J Am Coll Cardiol.

[8] Pijls NH, Klauss V, Siebert U, Powers E, Takazawa K, Fearon WF (2002). Coronary pressure measurement after stenting predicts adverse events at follow-up: a multicenter registry. Circulation.

[9] Klauss V, Erdin P, Rieber J, Leibig M, Stempfle HU, Konig A (2005). Fractional flow reserve for the prediction of cardiac events after coronary stent implantation: results of a multivariate analysis. Heart.

[10] Samady H, McDaniel M, Veledar E, De Bruyne B, Pijls NH, Fearon WF (2009). Baseline fractional flow reserve and stent diameter predict optimal post-stent fractional flow reserve and major adverse cardiac events after bare-metal stent deployment. JACC Cardiovasc Interv..

[11] Jensen LO, Thayssen P, Thuesen L, Hnsen HS, Lassen JF, Kelbaek LH (2007). Influence of a pressure gradient distal to implanted bare-metal stent on in-stent restenosis after percutaneous coronary intervention. Circulation.

[12] Matsuo A, Fujita H, Tanigaki T, Shimonaga T, Ueoka A, Tsubakimoto Y (2013). Clinical implications of coronary pressure measurement after stent implantation. Cardiovasc Interv Ther..

[13] Iwasaki K, Matsumoto T (2011). Coronary pressure measurement identifies patients with diffuse coronary disease who benefit from coronary revascularization. Coron Artery Dis.

[14] Hachamovitch R, Hayes SW, Friedman JD, Cohen I, Berman DS (2004). Stress myocardial perfusion single-photon emission computed tomography is clinically effective and cost effective in risk stratification of patients with a high likelihood of coronary artery disease (CAD) but no known CAD. J Am Coll Cardiol.

[15] Nijjer SS, Sen S, Petraco R, Escaned J, Echavarria-Pinto M, Broyd C (2014). Pre-angioplasty instantaneous wave-free ratio pullback provides virtual intervention and predicts hemodynamic outcome for serial lesions and diffuse coronary artery disease. JACC Cardiovasc Interv..

[16] Gould KL, Nakagawa Y, Nakagawa K, Sdringola S, Hess MJ, Haynie M (2000). Frequency and clinical implications of fluid dynamically significant diffuse coronary artery disease manifest as graded, longitudinal, base-to-apex myocardial perfusion abnormalities by noninvasive positron emission tomography. Circulation.

[17] De Bruyne B, Hersbach F, Pijls NH, Bartunek J, Bech JW, Heyndrickx GR (2001). Abnormal epicardial coronary resistance in patients with diffuse atherosclerosis but “Normal” coronary angiography. Circulation.

[18] Hachamovitch R, Hayes SW, Friedman JD, Cohen I, Berman DS (2003). Comparison of the short-term survival benefit associated with revascularization compared with medical therapy in patients with no prior coronary artery disease undergoing stress myocardial perfusion single photon emission computed tomography. Circulation.

[19] Farzaneh-Far A, Phillips HR, Shaw LK, Starr AZ, Fiuzat M, O’Connor CM (2012). Ischemia change in stable coronary artery disease is an independent predictor of death and myocardial infarction. JACC Cardiovasc Imaging..

[20] Kim YH, Ahn JM, Park DW, Song HG, Lee JY, Kim WJ (2012). Impact of ischemia-guided revascularization with myocardial perfusion imaging for patients with multivessel coronary disease. J Am Coll Cardiol.

[21] Ladenheim ML, Pollock BH, Rozanski A, Berman DS, Staniloff HM, Forrester JS (1986). Extent and severity of myocardial hypoperfusion as predictors of prognosis in patients with suspected coronary artery disease. J Am Coll Cardiol.

[22] Hachamovitch R, Berman DS, Kiat H, Cohen I, Cabico JA, Friedman J (1996). Exercise myocardial perfusion SPECT in patients without known coronary artery disease: incremental prognostic value and use in risk stratification. Circulation.

[23] Jahnke C, Nagel E, Gebker R, Kokocinski T, Kelle S, Manka R (2007). Prognostic value of cardiac magnetic resonance stress tests: adenosine stress perfusion and dobutamine stress wall motion imaging. Circulation.

[24] Schindler TH, Schelbert HR, Quercioli A, Dilsizian V (2010). Cardiac PET imaging for the detection and monitoring of coronary artery disease and microvascular health. JAAC Cardiovasc Imaging..

[25] Schindler TH, Schelbert HR, Quercioli A, Dilsizian V (2012). PET measurement of adenosine stimulated absolute myocardial blood flow for physiological assessment of the coronary circulation. J Nucl Cardiol..

[26] Massie BM, Hollenberg M, Wisneski JA, Go M, Gertz EW, Henderson S (1983). Scintigraphic quantification of myocardial ischemia: a new approach. Circulation.

[27] Costa MA, Shoemaker S, Futamatsu H, Klassen C, Angiolillo DJ, Nguyen M (2007). Quantitative magnetic resonance perfusion imaging detects anatomic and physiologic coronary artery disease as measured by coronary angiography and fractional flow reserve. J Am Coll Cardiol.

[28] Bruyne BD, Pijls NHJ, Heyndrickx GR, Hodeige D, Kirkeeide R, Gould KL (2000). Pressure-Derived Fractional Flow Reserve to Assess Serial Epicardial Stenoses Theoretical Basis and Animal Validation. Circulation.

